# Reprint of 'Model of unidirectional block formation leading to reentrant ventricular tachycardia in the infarct border zone of postinfarction canine hearts'

**DOI:** 10.1016/j.compbiomed.2015.08.013

**Published:** 2015-10-01

**Authors:** Edward J. Ciaccio, James Coromilas, Hiroshi Ashikaga, Daniel O. Cervantes, Andrew L. Wit, Nicholas S. Peters, Elliot R. McVeigh, Hasan Garan

**Affiliations:** a Division of Cardiology, Department of Medicine, Columbia University Medical Center, New York, United States; b Division of Cardiology, Department of Pharmacology, Columbia University Medical Center, New York, United States; c Division of Cardiovascular Diseases & Hypertension, Rutgers Robert Wood Johnson Medical School, New Brunswick, NJ, United States; d Division of Cardiology, Johns Hopkins University, Baltimore, MD, United States; e Department of Biomedical Engineering, Johns Hopkins University, Baltimore, MD, United States; f ARMGO Pharma, Inc., Tarrytown, NY, United States; g Myocardial Function Section, Imperial College and Imperial NHS Trust, London, United Kingdom

**Keywords:** Activation mapping, Isthmus, Unidirectional block, Ventricular tachycardia, Wavefront curvature

## Abstract

**Background:**

When the infarct border zone is stimulated prematurely, a unidirectional block line (UBL) can form and lead to double-loop (figure-of-eight) reentrant ventricular tachycardia (VT) with a central isthmus. The isthmus is composed of an entrance, center, and exit. It was hypothesized that for certain stimulus site locations and coupling intervals, the UBL would coincide with the isthmus entrance boundary, where infarct border zone thickness changes from thin-to-thick in the travel direction of the premature stimulus wavefront.

**Method:**

A quantitative model was developed to describe how thin-to-thick changes in the border zone result in critically convex wavefront curvature leading to conduction block, which is dependent upon coupling interval. The model was tested in 12 retrospectively analyzed postinfarction canine experiments. Electrical activation was mapped for premature stimulation and for the first reentrant VT cycle. The relationship of functional conduction block forming during premature stimulation to functional block during reentrant VT was quantified.

**Results:**

For an appropriately placed stimulus, in accord with model predictions: 1. The UBL and reentrant VT isthmus lateral boundaries overlapped (error: 4.8±5.7 mm). 2. The UBL leading edge coincided with the distal isthmus where the center-entrance boundary would be expected to occur. 3. The mean coupling interval was 164.6±11.0 ms during premature stimulation and 190.7±20.4 ms during the first reentrant VT cycle, in accord with model calculations, which resulted in critically convex wavefront curvature and functional conduction block, respectively, at the location of the isthmus entrance boundary and at the lateral isthmus edges.

**Discussion:**

Reentrant VT onset following premature stimulation can be explained by the presence of critically convex wavefront curvature and unidirectional block at the isthmus entrance boundary when the premature stimulation interval is sufficiently short. The double-loop reentrant circuit pattern is a consequence of wavefront bifurcation around this UBL followed by coalescence, and then impulse propagation through the isthmus. The wavefront is blocked from propagating laterally away from the isthmus by sharp increases in border zone thickness, which results in critically convex wavefront curvature at VT cycle lengths.

## Highlights

• Ventricular tachycardia is common after myocardial infarction.
• Reentrant ventricular tachycardia is often located in the infarct border zone.
• Conduction velocity of the leading edge of the propagating wavefront is not constant.
• Functional block occurs at locations where the wavefront becomes critically convex.
• Activation rate and IBZ geometric structure during reentrant ventricular tachycardia are determined.

## 1 Introduction

In postinfarction hearts, a reentrant circuit causing ventricular tachycardia (VT) can form within the infarct border zone, which is the thin region of remaining viable myocardium located between the infarct and the epicardial surface of the heart [1]. In canine postinfarction, a complete reentrant circuit is often apparent when the epicardial surface of the epicardial border zone is mapped with a multielectrode array [2]. In humans, post myocardial infarction reentrant VT circuits are mostly endocardial; however, epicardial circuits have also been described [3]. It has been shown that the isthmus, or diastolic pathway, present when the pattern of reentrant VT is that of a double-loop, coincides with the region of thinnest border zone [4–6]. Based in part on this observation, it was previously demonstrated that changes in activation wavefront curvature along the border zone *Z*-axis (thickness axis) likely result in very slow conduction or functional conduction block at the lateral isthmus boundaries during VT [6]. The wavefront becomes critically convex when it propagates in the outward direction at those locations due to the sharp spatial gradient from thinner to thicker border zone (lesser to greater volume of tissue activating), with a resulting source/sink mismatch. Because the current available forward to the propagation direction is insufficient to activate the larger volume of tissue downstream, the wavefront slows and blocks. Yet, block due to convex wavefront curvature is also activation rate dependent [7], and the relationship of activation rate to *Z*-axis thickness changes has not yet been considered.

More recently, it was shown that if the spatial change from thin to thick at the lateral isthmus boundaries has variable steepness, it can result in very slow and variable conduction across the lateral boundaries [8]. The resulting discontinuities in the laterally propagating electrical activation wavefront are a source of electrogram fractionation. Fractionation is defined as the presence of multiple variable deflections in the electrogram arising from asynchronous electrical activity. This compares with the normal condition in which a single activation wavefront is present, the source of the biphasic electrogram shape which is recorded from healthy myocardium. Because the geometric configuration of the conducting medium in postinfarction hearts is intransigent over time intervals of hours or more, electrogram fractionation can often be observed at the same recording location during normal sinus rhythm as well as during reentrant VT, depending on the wavefront direction [8] and the coupling interval for electrical activation.

Although the functional block lines forming at the lateral isthmus boundaries, and electrogram fractionation arising from those locations, can be explained on the basis of critically convex wavefront curvature, the cause of unidirectional block line (UBL) formation during premature stimulation, which leads to onset of reentrant VT with a double-loop reentrant circuit pattern, has not been entirely elucidated. It is possible that large spatial changes in refractoriness are partially responsible for UBL formation leading to reentrant VT, as was shown in some prior canine experiments [9,10]. However in canine postinfarction, the effective refractory period at the isthmus location (inner pathway), versus immediately outside the isthmus (outer pathway), may also have little or no difference [11,12]. A low safety factor associated with longitudinal propagation in a non-uniformly anisotropic substrate can also contribute to conduction block [13]. Alternatively, herein it is shown how critically convex wavefront curvature, caused by changes in infarct border zone thickness, can result in unidirectional conduction block during premature excitation, and lead to the formation of a double-loop reentrant circuit with central isthmus at VT onset. The model can be helpful for understanding reentrant circuit formation because wavefront curvature changes have an anatomical, i.e., structural basis, and do not require the presence of sharp spatial changes in molecular-level properties at locations where functional block lines arise.

## 2 Method

### 2.1 Canine data acquisition

**Fig. 1:**
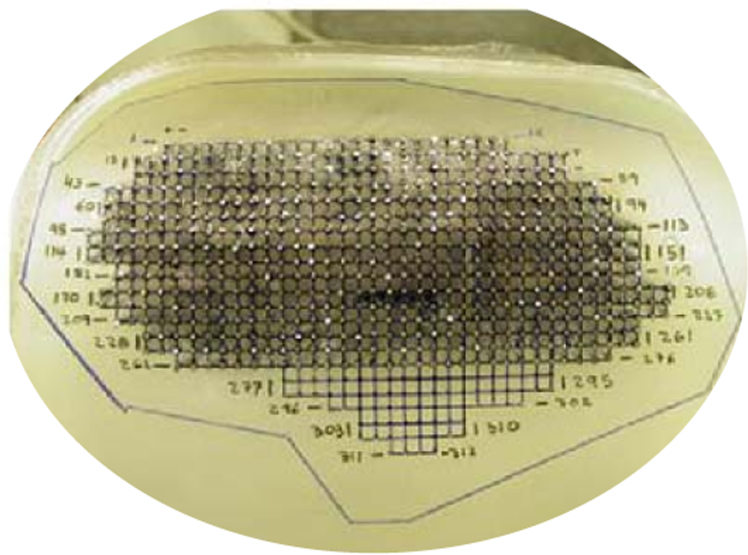
Multielectrode array during its construction. Electrodes are silver disks approximately 1 mm in diameter on a square grid, embedded in a polyurethane material. The writing notes the bipolar electrode numbers in each row. The edges of the array that will be cut from the polyurethane material are delineated in dark blue. (For interpretation of the references to color in this figure legend, the reader is referred to the web version of this article.)

From an archived retrospective database of canine postinfarction, experiments were selected for analysis in which monomorphic ventricular tachycardia with a stable double-loop reentrant circuit was inducible by premature stimulation. Of 20 experiments that were analyzed, 12 met the inclusion criteria for the study. In all experiments, mongrel canines weighing 20–40 kg were anesthetized using sodium pentobarbital (intravenous, 30 mg/kg). The left anterior descending coronary artery (LAD) was then ligated near its base [1,2]. A transmural anteroseptal myocardial infarction resulted, with an epicardial rim of muscle having variable thickness, termed the infarct border zone. Canines were prepared for electrophysiological analysis 3–5 days following ligation of the LAD, which is the interval during which the postinfarction canine heart is most likely to be arrhythmogenic [1,2,4,5,14]. Bipolar electrodes sutured onto the left ventricle, near the LAD artery, at the base of the heart (BASE), the center region, and the left lateral edge (LAT), were used to induce reentrant VT via programmed electrical stimulation. A train of 10 S1 pulses with an interval of 250–300 ms between pulses was followed by a premature stimulus. The pace train was repeated, shortening the last stimulus interval by 5–10 ms on each subsequent train, until reentry was initiated or until block occurred at the stimulus site. During the normal sinus rhythm, premature stimulation, and during any monomorphic VT that was induced, electrograms were simultaneously recorded from the epicardial surface of the left ventricle, where the border zone usually forms in postinfarction canine hearts after LAD ligation, from 196 to 312 bipolar electrodes configured as a multielectrode array. These methods were previously described in detail [2]. An image of the 312 bipolar multielectrode array taken during its construction is shown in Fig. 1. The array consists of silver disks 1 mm in diameter arranged on a square grid and embedded in polyurethane. Each silver disk was formed by melting the end of a thin solid silver wire. The 624 silver wires (two for each of 312 bipolar pairs) were attached to the data acquisition system via a zero insertion force (ZIF) connector. At the data acquisition system, a differential amplifier was used to form bipolar signals from pairs of leads, which were bandpass filtered at 0.5–500 Hz, digitized at 1 to 2 kHz per channel, and streamed to computer disk. Further details of the electronic configuration of the acquisition system and data throughput are described elsewhere [15], as are details of electrical activation mapping [2,4,5,14]. The quantitative methods for registration of the multielectrode array with respect to anatomical landmarks have also been described [16]. The canine experiments were approved by the Institutional Review Board of Columbia University Medical Center, and the experimental paradigm was in accord with institutional guidelines.

### 2.2 Model of unidirectional block leading to reentrant VT

In prior work, details concerning the relationship between infarct border zone thickness, wavefront curvature, and conduction velocity were provided [6]. In this section it is shown how the wavefront curvature model can be used to predict where functional electrical conduction block will form in the infarct border zone. The summary equation for thickness-induced changes in wavefront curvature in the border zone is given by [6]:

(1) $$\theta \approx \theta_{0}-\frac{D}{c}\cdot \frac{\Delta T}{T}$$

where *θ* is the conduction velocity of the propagating wavefront, *θ*_0_ is the conduction velocity of the propagating wavefront when the geometry of the conducting medium along a particular distance is unchanged, resulting in a rectilinear (straight) leading edge (~0.4 mm/ms in ventricular myocardium), *T* is the border zone thickness, *c* the space step and *D* the diffusion coefficient are constants, and Δ*T* is the spatial change in thickness per unit space step *c*, taken as the absolute maximum Δ*T* in the vector field about the measurement point. For the present study, values of *c*=1 mm or unity (cia07) and *D*=0.1 mm^2^/ms [17] were used. Based on Eq. (1), therefore, when Δ*T*/*T*→4.0 (unitless), *θ*→0 mm/ms, i.e., wavefront curvature becomes critically convex. This will occur when *T* is minimized and Δ*T* is maximized when the activation wavefront propagates from thin to thick infarct border zone. The latter condition is met at the lateral boundaries of the reentry isthmus, since the isthmus is coincident with the thinnest border zone, and since it has been shown that there is a maximal change to thicker border zone outward in the lateral directions [6].

As a first approximation, suppose that the effective refractory period of the myocardial tissue is relatively uniform throughout the infarct border zone, and for simplicity, suppose conduction, as a first approximation, is isotropic. The likelihood of conduction block for a given level of wavefront convexity is rate dependent [7]. When the premature stimulus interval is shorter than the cycle length of reentrant VT, block is more likely to occur at a given level of wavefront curvature during premature stimulation as compared with during VT.

**Fig. 2:**
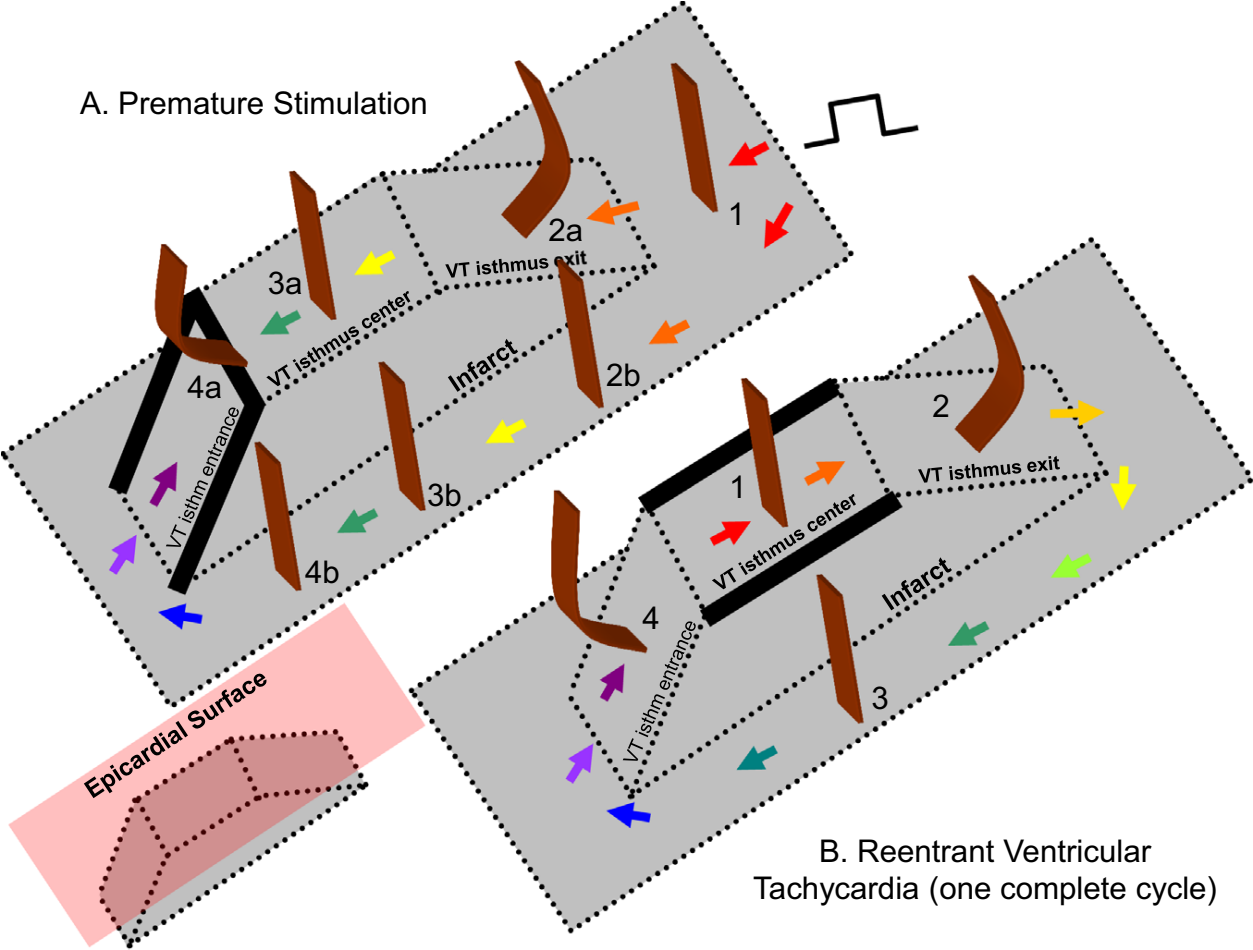
Schematic of block line formation dependent upon infarct border zone thickness. A. during premature stimulation. B. during reentrant ventricular tachycardia. (For interpretation of the references to color in this figure, the reader is referred to the web version of this article.)

Based on Eq. (1) and the relationship of cycle length to block, it would be expected that alterations in wavefront curvature are as noted in Fig. 2. For the schematics of both premature stimulation (panel A) and ventricular tachycardia (panel B), the infarct is shown in gray, and wavefront curvature is noted in brown. As a reference, in the inset at lower left, the epicardial surface is depicted as a red plate (not present in panels A and B for clarity of detail). To show sequence progression over time, short arrows depict the direction of propagation from red to violet in panels A and B. During premature stimulation (panel A), the wavefront is rectilinear near the stimulus site, where there is no change in border zone thickness (labeled 1). As the wavefront encounters the isthmus location, where the border zone is thinner, it becomes concave, facilitating propagation (2a). Propagation outside the isthmus in the same direction is rectilinear (2b), since there is no change in thickness in the propagation direction there. For simplicity, propagation outside the isthmus is only shown on one side. Propagation along the isthmus center, and also outside the isthmus location in the same direction is rectilinear (3a, 3b), since there is no change in thickness in the propagation direction. As the wavefront encounters a change to thicker tissue at the isthmus end (4a) it becomes convex, slowing conduction. At the short coupling interval during premature stimulation, conduction blocks at this degree of curvature, noted by thick black lines at the isthmus center-entrance boundary location and at the lateral edges of the isthmus entrance location. However, block does not occur at 4b, because there is no change in thickness in the propagation direction. From 4b, the wavefront curves around. It cannot propagate directly from 4b to 4a due to the discontinuity that would result from wavefront pivoting [19]. Were the wavefront to move obliquely across the isthmus entrance boundary from 4b, it would slow or block, since the transition in the forward direction within the entrance area would be thin-to-thick. Toward the isthmus end, the wavefront coalesces with the wavefront arriving from the other side of the isthmus (not shown), activates the isthmus entrance, and reenters the isthmus center in the opposite direction, which has recovered excitability. During a full cycle of reentrant VT, the configuration is as shown in panel B of Fig. 2. Propagation through the isthmus center is rectilinear because there is approximately no change in thickness (1). However, were portions of the wavefront to propagate toward the lateral isthmus boundaries, they would block due to the steep lateral transition from thin-to-thick border zone there. As the wavefront encounters the isthmus exit, it becomes convex because of the thin-to-thick transition (2). However, because cycle length during stable monomorphic reentrant VT is longer than during premature stimulation, critically convex curvature does not occur and it does not block. The wavefront bifurcates at the isthmus exit and then propagates around the outer pathway as a rectilinear wavefront (3). Propagation at the isthmus entrance is facilitated because of the thick-to-thin change in border zone thickness, resulting in concave wavefront curvature (4). Thus there is a change in wavefront propagation direction only at isthmus center and exit locations during premature stimulation (panel A) versus reentrant VT (panel B).

**Fig. 3:**
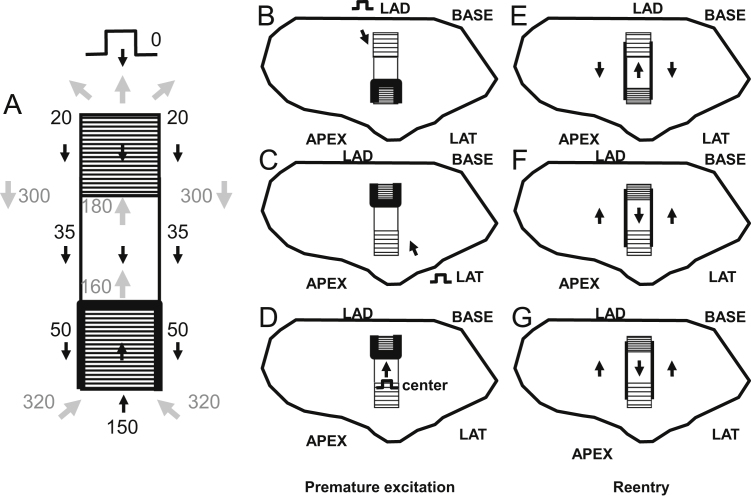
Schematic of the timing during the premature stimulus and during the first reentrant ventricular tachycardia cycle (A), stimulus sites for unidirectional block line formation at either isthmus end (B–D), and direction of propagation during reentrant ventricular tachycardia, shown by arrows (E–G).

Based on the above paradigm, the timing relationship of the premature stimulation cycle versus the first reentrant VT cycle is depicted in Fig. 3A. Activation wavefront propagation direction during the premature excitation cycle is denoted with black arrows. Examples of approximate timing are given, with the actual timing depending in part on the distances between landmarks. The premature excitation wavefront arrives at time 50 ms at the opposite end of the isthmus and blocks around it on three sides due to the steep thin-to-thick transition in the forward wavefront direction. Toward the end of the premature stimulation cycle, the bifurcated wavefronts coalesce, and are facilitated to rapidly propagate in the travel direction at time 150 ms since the spatial transition is thick-to-thin. A single impulse then proceeds and enters the isthmus. Due in part to the wavefront curving a total of 180° in this region, there is a long delay for arrival at the reentry point (from time 50 ms to time 160 ms). This is a consequence of propagation of the wave about a pivot point, with convex wavefront curvature slowing the conduction velocity about such points [18,19]. A gray arrow shows the propagation direction as the wavefront breaks through the unidirectional block line on the opposite side to initiate the first reentry cycle (time 160 ms). The wavefront proceeds through the isthmus center (time 160 to 180 ms). It is constrained in this region as a narrow electrical impulse, and is prevented from propagating outwardly across the lateral boundaries due to the steep change in thin-to-thick transition there (see also Fig. 2B). The impulse then proceeds through the isthmus exit. There is a slight possibility of block at short VT cycle lengths at this location due to convex wavefront curvature, depending on the shortness of the cycle length. However, if the wavefront does propagate through the exit, it then bifurcates, the distinct wavefronts slowly pivot, then travel in the downward direction in the schematic away from the lateral boundaries (gray arrows, time 300 ms), and arrive at the isthmus entrance (time 320 ms), whereupon they coalesce, completing the first reentry cycle. Thus the double-loop pattern of reentry is established during premature stimulation and the first reentry cycle, with a VT cycle length of approximately 200 ms on average [20]. On subsequent cycles, due to the constraints of thin-to-thick transition and because of the refractory relationships forming during the first cycle, the pattern perpetuates, so long as wavefront breakthrough does not occur.

The isthmus end with sharpest spatial transition Δ*T*/*c* would be anticipated to most likely block from an appropriately placed premature stimulus, as convex wavefront curvature will be greater and closer to the critical value there. This is illustrated in Fig. 3B–D. The isthmus ends are shown having a more gradual slope (lesser density of horizontal lines and longer path) or a steeper slope (greater density of horizontal lines and shorter path to effect the same change in thickness). The configurations that are schematized in Fig. 3B–D would occur in different postinfarction hearts. In Fig. 3B, the premature excitation wavefront originating from the LAD blocks on the opposite end of the isthmus from the stimulus site due to the sharp thin-to-thick transition there (similarly when the stimulus is from the BASE – not shown). In Fig. 3C–D, the premature excitation wavefront originating from the LAT or from the center, respectively, also block at the opposite end of the isthmus from the stimulus site due to the sharp thin-to-thick transition. The corresponding activation patterns during reentry, for the premature stimulation patterns of Fig. 3B–D, are shown in Fig. 3E–G. The isthmus entrance is located at the end with steepest spatial change in thickness. The isthmus exit is located at the end having the most gradual spatial change in thickness.

At locations where wavefront curvature approaches a critical convexity, when the rate of activation is increased, the likelihood of conduction block also increases [7]. To show the rate dependency relationship, the conduction velocity equation can be written as [19,21]:

(2) $$\theta =\theta_{0}-D\cdot \rho$$

where *ρ* is the absolute magnitude of the degree of wavefront curvature. From Eq.'s (1) and (2):

(3) $$\rho =\frac{1}{c}\cdot \frac{\Delta T}{T}$$

The conduction velocity equation can also be modeled as a circular arc [22]:

(4) $$\theta =\theta_{0}-D\cdot \frac{2\cdot \sin\;(\beta )}{w}$$

where *β* is the angle between the propagation direction and the taper of the conducting medium, *w* is the distance across the conducting medium at the wavefront leading edge, and:

(5) $$\rho =\frac{2\cdot \sin\;(\beta )}{w}$$

Based on a prior analysis of reentrant VT in postinfarction canine hearts [6], mean border zone thickness *T* was shown to average 231 μm at the isthmus location and 1440 μm along the outer pathway of the circuit. Thus the average change in thickness between isthmus and outer pathway is Δ*T*=1440−231=1209 μm, and:

(6) $$\frac{\Delta T}{T}=\frac{1440-\text{231}\;}{231}=5.23\;\left(\mathrm{unitless}\right)$$

in the direction from thinnest isthmus to thicker outer pathway. If Δ*T* occurs over a spatial interval of *c*=1 mm, then from Eq. (1), this would likely result in conduction block, since the value of Δ*T*/*T* is greater than 4.0. However, the occurrence of conduction block when wavefront convexity approaches a critical value of curvature also depends upon activation rate. When a narrow isthmus opens to an expanse, the angle *β*=90°, and conduction block has been shown to occur at *w*≤0.6 mm for a rate of 200 ms [7].The interval 200 ms is approximately the mean reentrant VT cycle length as induced by premature stimulation in canine postinfarction experiments [20]. At this value of *w*:

(7) $$\rho =\frac{2\cdot \sin\;(90{}^{\circ})}{0.6\;\mathrm{mm}}=3.33\;{\mathrm{mm}}^{-1}$$

From Eq. (3), the distance interval, or space step *c*, corresponding to this value of *ρ* is thus:

(8) $$c=\frac{\Delta T}{T\cdot \rho }=\frac{5.23}{3.33\;{\mathrm{mm}}^{-1}}=1.6\;\mathrm{mm}$$

Based upon Eq. (8), the spatial distance over which the thickness transition from 231 μm to 1440 μm should occur in order to result in block across a lateral boundary would be ~2 mm, which can be considered to be a steep spatial change. This is in accord with the actual distance over which *ρ* is maximal across the lateral isthmus boundaries, on the order of 2 mm as is evident in a prior study [6].

Consider now the occurrence of conduction block for an activation interval of 150 ms, which is approximately the value of the premature stimulation coupling interval used for VT induction in canine postinfarction experiments [2]. Block through a narrow isthmus occurs when *w*≤1.29 mm [7]. At this value of *w*, the degree of wavefront curvature is:

(9) $$\rho =\frac{2\cdot \sin\;(90{}^{\circ})}{1\mathrm{.29}\;\mathrm{mm}}=1\mathrm{.55}\;{\mathrm{mm}}^{-1}$$

From Eq. (3), the distance interval, or space step *c*, for this value of *ρ* is:

(10) $$c=\frac{\Delta T}{T\cdot \rho }=\frac{5.23}{1.55\;{\mathrm{mm}}^{-1}}=3.4\;\mathrm{mm}$$

Moreover, at a very fast rate of 117 ms, which is occasionally an approximate premature coupling interval for which induction of VT in canine postinfarction results, conduction block was shown to occur when *w*≤2.64 mm [7]. The degree of wavefront curvature is then:

(11) $$\rho =\frac{2\cdot \sin\;(90{}^{\circ})}{2.64\;\mathrm{mm}}=0\mathrm{.758}\;{\mathrm{mm}}^{-1}$$

The distance interval, or space step *c*, for this value of *ρ* is:

(12) $$c=\frac{\Delta T}{T\cdot \rho }=\frac{5.23}{0\mathrm{.758}\;{\mathrm{mm}}^{-1}}=6.9\;\mathrm{mm}$$

Based upon the result of Eq.'s (10) and (12), it would be anticipated that block at an isthmus end would occur during premature excitation when the distance over which a thickness transition from 231 to 1440 μm occurs is in the range of approximately 5–10 mm. Therefore on average, it would be expected that the UBL described in Figs. 1 and 2 would extend for a distance of approximately 5–10 mm, beginning at the isthmus center-entrance boundary. These model estimates of conduction block parameters were tested as described below.

### 2.3 Quantitative analysis to test the model

**Fig. 4:**
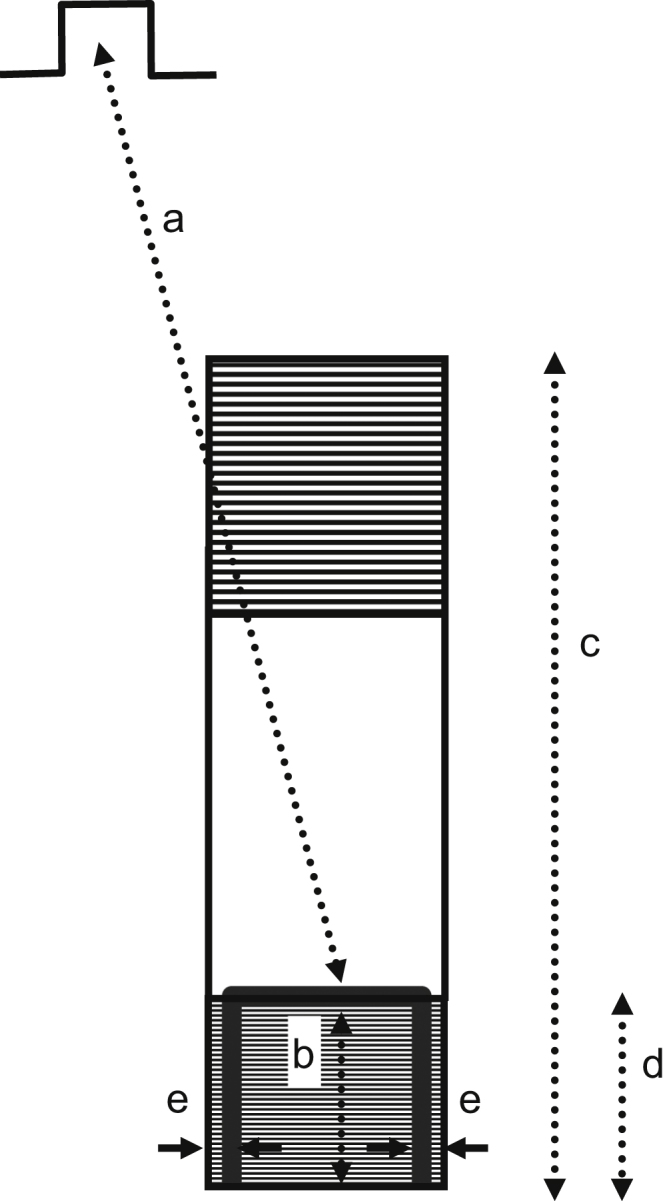
Schematic of the measurements made to show the relationships between stimulus site, unidirectional block line forming during premature stimulation, and reentrant ventricular tachycardia isthmus. The stimulus site is noted at top left. The reentrant circuit isthmus is depicted as a rectangular surface. It is hatched at either end to denote areas where sharp changes in infarct border zone thickness occur. The location where unidirectional block forms during premature stimulation is noted as a thick curved black line. It approximately coincides with the boundaries of what will be the entrance portion of the reentry isthmus during ventricular tachycardia.

For all experiments, the location of the UBL leading to reentrant VT was determined by activation mapping of the premature stimulation cycle. The location of the reentrant circuit isthmus, and the direction of propagation through the isthmus, was measured during the first complete cycle of reentrant VT, and during a subsequent cycle within 10 beats as a check of the stability of the circuit. On a computerized grid, the unidirectional block position as delineated by a curved line was overlaid on the isthmus location. The stimulus site location was also marked. It was determined whether the UBL of premature stimulation coincided with the isthmus entrance boundary of VT, based upon the following measurements (Fig. 4). Length *a*: the distance from the stimulus site to the leading edge of the UBL. Length *b*: the distance over which the UBL extends. Length *c*: the isthmus length along its long axis, averaged if the two lateral block lines have disparate lengths. Value of length *d*, the projection of the UBL lateral edges onto the locations of the VT lateral block lines, divided by isthmus length *c*, ratio *d*/*c*: the fraction of the total isthmus length that is overlapped by the UBL, expressed as a percent. Length *e*: the closest distance between the lateral edges of the UBL and the lateral isthmus boundary, averaged from both sides, which was used as an error function.

For all 12 experiments, the value of the premature coupling interval leading to reentrant VT, and the mean cycle length of VT at onset (first 10 cycles) were also tabulated. At the site of breakthrough initiating reentry, the interval between activation during premature stimulation and activation during the first reentrant VT cycle, termed the reexcitation interval, was recorded.

## 3 Results

**Fig. 5:**
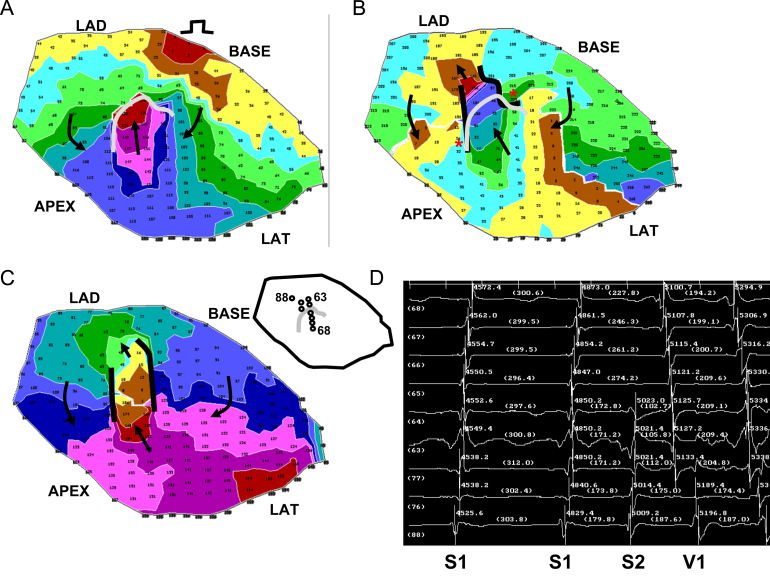
Activation map of the premature stimulus cycle from the BASE, the first reentrant ventricular tachycardia cycle, a subsequent reentrant ventricular tachycardia cycle, and the electrograms recorded at the isthmus region at the time of reentry onset. (For interpretation of the references to color in this figure, the reader is referred to the web version of this article.)

In Fig. 5 is shown a typical result, for experiment 7. The view is of the epicardial surface of the anterior left ventricle, where the infarct border zone is located. The activation map for the premature stimulation cycle leading to reentrant VT is shown in panel A. The stimulus location is noted near the BASE. Isochronal colors denote areas with activation times differing by 15 ms. The UBL is shown in gray. Its leading edge is toward the stimulus site, and the activation wave bifurcates around it, beginning at time ~70 ms into the cycle (arrows). Due to the curvature of the wavefront around each side of the UBL, and considering that propagation about a pivot point fails beyond a critical value of curvature [19], propagation is delayed.

The two wavefronts begin to coalesce at time ~110 ms (violet isochrones) and then proceed to the opposite side of the UBL, arriving at time 180 ms (arrow pointing toward the LAD). Whereupon, time for recovery of excitability, which is 180 ms – 74 ms=103 ms (panel A), is sufficient so that the wavefront breaks through and reenters the previously excited region to establish the first reentry cycle (panel B, arrow closest to the LAD). The functional lines of block at the lateral boundaries of the isthmus during the first reentrant VT cycle are shown as black curved lines, and arrows point out the activation sequence (panel B). For reference, the UBL of premature excitation is shown overlaid on the grid in gray color. There is an approximate coincidence between the locations of the lateral edges of the UBL and the lateral boundaries of the reentry isthmus during VT (red asterisks in panel B). The area of unidirectional block during premature stimulation approximately coincides with the distal half of the isthmus location, which serves as the entrance to the isthmus during reentrant VT. In panel C is shown the reentry activation map during a subsequent cycle (cycle 10) when the circuit has more completely stabilized; it is mostly similar to the first reentry cycle in terms of length and orientation, as well as activation sequence (arrows). In panel D, electrograms are shown which include the premature stimulus cycle with a coupling interval of 170 ms, and the first reentry cycle (V1), as noted. In the inset at upper left of panel D, the locations of the recorded electrograms are shown with respect to the UBL, with channels 88, 76, 77, 63, and 64 being on the LAD side, proximal to the UBL, and channels 65–68 being distal to this block line. During the S2 cycle, block occurs between channel 64 and 65 (panel D), which corresponds to the recording sites on opposite sides of the UBL leading edge (inset). The distinct activation wavelets proceed around and coalesce on the opposite side of the UBL, so that the activation sequence is in the reverse direction during V1. There is some delay in activation at the exit sites (76, 75, and 88) as was predicted by the model, due to the likelihood of *Z*-axis convex curvature there, resulting in slowed wavefront propagation. The reexcitation interval of 103 ms (time for reactivation at site 64 from S2 to V1) is substantially shorter than the S1–S2 coupling interval of 170 ms.

**Fig. 6:**
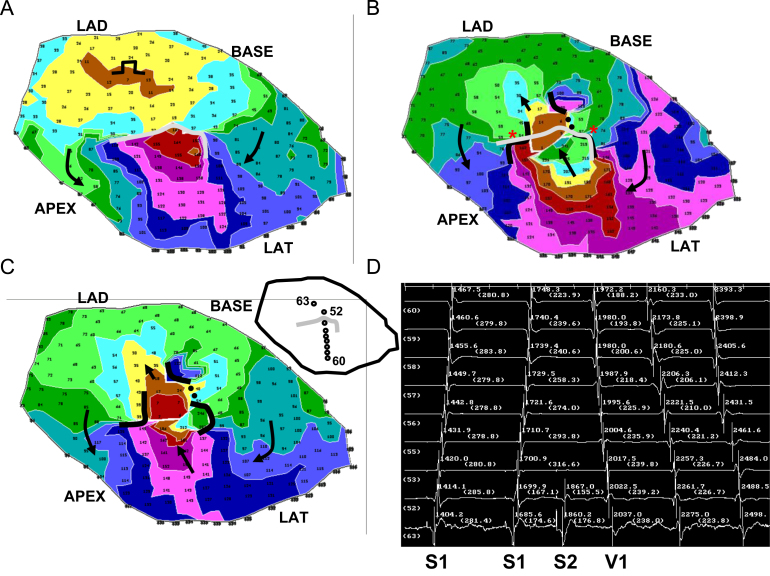
Activation map of the premature stimulus cycle from the LAD region, the first reentrant ventricular tachycardia cycle, a subsequent reentrant ventricular tachycardia cycle, and the electrograms recorded at the isthmus region at the time of reentry onset.

A second example of reentry induction after a short S1–S2 coupling interval is shown in Fig. 6 for experiment 9, with the stimulus site being located toward the LAD (panel A). The UBL of premature stimulation is again shown as a curved gray line in panel A, while functional block at the lateral isthmus boundaries during the first cycle of reentrant VT are denoted by curved black lines in panels B and C. There is much overlap of the block lines (asterisks in panel B), with the UBL, shown overlapped in gray, coinciding with the distal half of the isthmus location. The electrogram recordings (panel D) show a short reexcitation interval of 156 ms (channel 52) as compared with the S1–S2 coupling interval (175 ms). The electrode locations are noted in the inset.

**Fig. 7:**
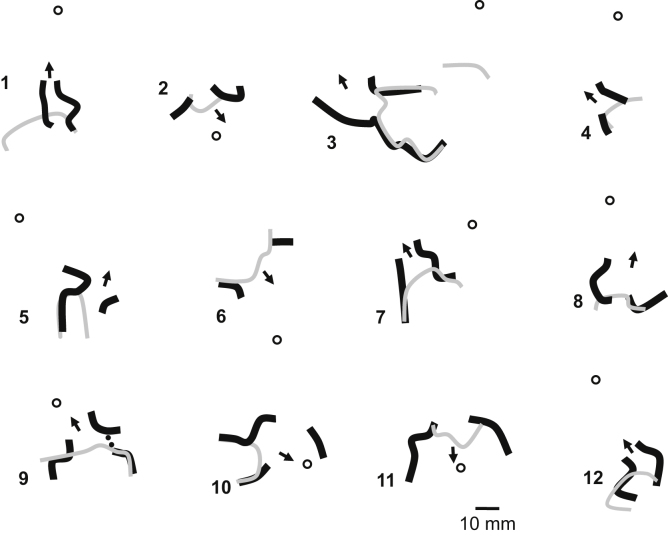
Comparison of the locations of the premature stimulus site (circle), the unidirectional block line forming from this site (thinner curved gray line), and functional block (thick curved black lines) during double-loop reentrant ventricular tachycardia, for all 12 experiments of the study. In each panel, the arrow denotes the direction of propagation through the isthmus during reentrant ventricular tachycardia. In panel 9, very slow conduction rather than actual block is denoted by a dotted line.

In Fig. 7 is depicted the results of activation mapping for all 12 postinfarction canine experiments, which are numbered. A marker depicting 10 mm in length is shown at lower right in the figure, and it applies to all 12 panels, which are drawn to scale. The LAD side of the grid is at top for all 12 panels. Functional lines of conduction block during reentry are illustrated as thick black lines. The UBL of premature stimulation resulting in reentry is delineated as a thinner gray line. The stimulus site is noted by a circle, and the direction of propagation leading to breakthrough at onset of the first reentry cycle is delineated as a black arrow. The stimulus site is approximately in the direction of this arrow, in proximity to the isthmus exit location. The UBL forms at the isthmus end that is furthest from the stimulus site, which becomes the isthmus entrance during reentry. The lateral UBL edges approximately coincide with the lateral isthmus boundaries during reentry. Overall, the error was 4.8±5.7 mm (measurement *e*, Fig. 3). The relatively large standard deviation occurred because in six experiments the UBL edges and the lateral isthmus boundaries overlapped (error=0) while in a few experiments there was some misalignment.

The measurements done for each experiment of Fig. 7 are shown in Table 1. Pooled for all 12 experiments, the mean distance from stimulus site to the UBL leading edge was 31.4±14.2 mm (measurement *a*, Fig. 3). The mean length of the UBL lateral edges was 10.7±5.7 mm (measurement *b*, Fig. 3). This is approximately in accord with the range of 5–10 mm predicted by the model. The mean isthmus length along the long axis was 23.1±9.3 mm (measurement *c*, Fig. 3), and is in accord with prior observations [20]. The mean length of the UBL projected along the isthmus long axis was 8.9±4.5 mm (measurement *d*, Fig. 3). Measurement *d* averaged slightly less than measurement *b* due to misalignment between the UBL lateral edges and the lateral block lines of VT. On average, the leading edge of the unidirectional block line began at a point 38.5±9.7% of the way along the isthmus with respect to its distal end (percent of measurement *d*/measurement *c*), as would be anticipated for block occurring at the isthmus center-entrance boundary. In Table 2, values for the premature coupling interval and the first reentrant VT cycle length are shown. The mean value for premature coupling interval for all 12 experiments was 164.6±11.0 ms while for VT cycle length it was 190.7±20.4 ms. These actual mean values for premature stimulation and VT are in agreement with those used for model calculations to support conduction block (Eq.'s (6)–(12) and Fig. 2). Thus unidirectional block at the isthmus entrance location is supported during the premature stimulation cycle, and functional block at the lateral isthmus boundaries is supported during reentrant VT cycles. The reexcitation interval (time difference for activation during premature stimulation versus cycle 1 of VT at the breakthrough point) averaged 150.9±26.7 ms for the 12 experiments; in 8/12 experiments the reexcitation interval was shorter than the premature coupling interval.

## 4 Discussion

### 4.1 Summary

In this study a model of UBL formation during premature stimulation, leading to reentrant VT with a double-loop circuit, was described for canine postinfarction. The model is based on changes in activation wavefront curvature along the infarct border zone *Z*-axis (thickness axis). It was shown in a series of 12 experiments that the lateral edges of the UBL forming during premature stimulation which results in reentrant VT tend to coincide with portions of the lateral isthmus edges (Figs. 5–7). Conduction block at the isthmus entrance location was correctly predicted to be supported when the premature coupling interval was on the order of 117–150 ms. During the premature stimulus cycle, when unidirectional block occurred around the isthmus entrance location, as predicted by the model, the wavefront would then bifurcate, curve around with delay, coalesce, and reenter the previously excited region. Conduction block at the lateral isthmus edges during reentrant VT was correctly predicted to be supported for a mean cycle length of 200 ms.

### 4.2 Consequences of the model

Onset of reentry can only occur when the isthmus entrance region, which is the area of steepest thin-to-thick transition in the direction of wavefront propagation during premature stimulation (Fig. 3B), is sufficiently steep for a particular short premature coupling interval so that critical wavefront convexity resulting in block can occur, and of sufficient size to support the delay necessary for recovery of excitability and breakthrough into the previously excited region, initiating the first reentry cycle (Figs. 2 and 3). Although there is a thin-to-thick transition at the isthmus exit during reentry, block there would not be expected to occur due to the relatively gradual transition, unless a short VT cycle length resulted from wavefront breakthrough elsewhere in the circuit, causing critical wavefront convexity there. Reentrant VT would not be expected to be inducible by stimulating from the APEX or LAT in Fig. 3B, nor from the LAD or BASE in Fig. 3C and D. If however both isthmus ends were to have a sufficiently large and sharp transition region, depending on the stimulus site location, either might possibly act as points to initiate reentry, i.e., there would be two possible reentry morphologies. However, the likelihood of block at the isthmus exit for either of these VT circuit morphologies would presumably be increased, due to the relatively steep transition at whichever isthmus end serves as the exit. Thus any induced VT episodes would probably be short-lived. Future studies are needed to test this hypothesis.

Based on the model, refractoriness within the infarct border zone is important for initiation and maintenance of reentry, in the sense that the UBL must be of sufficient size so that there is recovered excitability when the wavefront arrives on the opposite side at the end of the premature stimulus cycle. Furthermore, depending in part upon cycle length (see Eq.'s (6)–(8)), if very slow conduction rather than actual block occurs when the activation wavefront propagates outward across the lateral isthmus boundaries during the diastolic interval of reentry, as proposed elsewhere [8], refractoriness at the outer portion of the circuit may be necessary to prevent wavefront breakthrough and thus to maintain reentry.

## 5 Conclusions

**Fig. 8:**
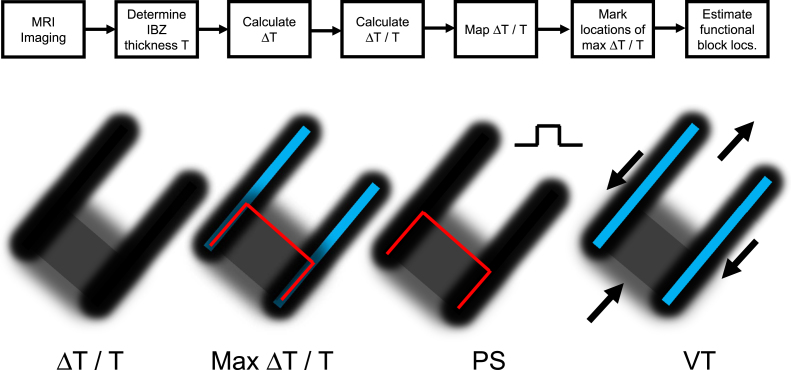
Top: set of steps that can be used to estimate functional block line locations during premature stimulation and during reentrant ventricular tachycardia. Δ*T* is taken as the largest absolute value of Δ*T* in the vector field about the measurement point. Lower: Maps of Δ*T*/*T*, maximum Δ*T*/*T* (shown as red – during premature stimulation and blue – during ventricular tachycardia), block line locations during premature stimulation (PS) and during ventricular tachycardia (VT). For simplicity, an area of large Δ*T*/*T* is shown at only one of the two isthmus ends. (For interpretation of the references to color in this figure legend, the reader is referred to the web version of this article.)

Based on the quantitative model described herein, functional block line locations can be estimated after measurement of infarct border zone thickness, for example by using enhanced magnetic resonance imaging or MRI (Fig. 8, top). Mapping Δ*T*/*T* and detecting areas of maximal absolute change in Δ*T*/*T* are key to the estimation process (Fig. 8, lower left panels). In instances when the premature excitation cycle leads to a stable reentrant VT circuit pattern initiated on the next cycle, the characteristics of wavefront curvature due to changes in border zone thickness along the *Z*-axis are useful to describe the electrophysiologic events that occur. When the premature coupling interval during programmed electrical stimulation is sufficiently short, conduction blocks unidirectionally along the surface area with steepest thin-to-thick transition, which corresponds to the isthmus entrance during reentrant VT (Fig. 8, PS). The UBL tends to coincide with the isthmus entrance boundary on three sides, where Δ*T*/*T* would be largest since *T* is minimized there and Δ*T* changes from thin-to-thick in the direction of wavefront propagation (Eq. (1)). Due to the bifurcation and curving of the premature stimulation wavefront around the UBL, followed by coalescence, and then by breakthrough into the previously excited region, with the constraint that the electrical impulse within the isthmus blocks in the direction of the lateral boundaries, the pattern of activation during subsequent reentry cycles with a double-loop circuit is established (Fig. 8, VT panel). This double-loop circuit pattern with central common pathway is often observed in canine postinfarction, and also in clinical cases of reentrant VT [23].

### 5.1 Limitations and future directions

The quantitative model described in this study cannot be used to predict the reentrant circuit activation pattern when VT onset is unstable. In previous work, reentry episodes with unstable onsets were not excluded, resulting in more varied locational relationships between the UBL and isthmus location [24]. In some cases when VT onset is unstable, the UBL can form at the isthmus end that is most proximal to the stimulus site, or even proximal to the isthmus location itself [6,24].

The precise rate dependency of conduction block occurring at the isthmus entrance, with respect to the steepness of the thickness transition, is unknown and should be determined in future experiments. Furthermore, experiments with magnetic resonance imaging or histological analysis are needed to determine whether the UBL leading edge coincides exactly with the location of the isthmus center-entrance boundary as defined in Fig. 2. To develop the model described herein, isotropic conduction was supposed. The effect of anisotropic conduction in delaying propagation in the direction transverse to muscle fibers [25], as well as the effect of safety factor on myocardial conduction [13], should be considered for further development of the model. Propagation through constricted regions along the XY plane (i.e., the surface plane of the heart) [26] can also lead to critically convex wavefront curvature, and may be responsible for some of the slowing and block that was observed in this series of experiments. Further development of the model should include wavefront curvature in the XY plane as well as along the *Z*-axis.

### 5.2 Clinical perspective

VT caused by a reentrant circuit can occur in myocardial infarction patients and is a major public health concern. Untreated, it can lead to sudden cardia death. To treat the arrhythmia, radiofrequency ablation is often used to destroy arrhythmogenic tissue. A common clinical problem is that the best region to ablate the heart to interrupt the circuit and prevent reinduction of reentrant VT is difficult to ascertain. This can be due to the fact that during patient electrophysiologic study, it may not be possible to induce the clinical tachycardia so that it can be characterized. Even if the clinical tachycardia is inducible, the patient can be hemodynamically compromised and will not tolerate the induction of tachycardia well; thus it must be terminated prior to characterization. The model described herein suggests a mechanism by which functional electrical conduction block forms during premature stimulation and during reentrant VT. Since, according to the model, block depends on geometric changes in the structure of the infarct border zone, the narrow region of surviving tissue in proximity to the infarct, imaging methods with sufficient spatial resolution, notably cardiac magnetic resonance imaging, can be used to determine the structural configuration, for estimation of functional block locations. Other types of imaging methods coming online may also soon be capable of detailing the heart structure with sufficient resolution to determine structural configuration and functional block. Once functional block locations are known, radiofrequency ablation across the narrowest isthmus width would likely prevent reinduction of the reentrant VT morphology, since impulse propagation is the most constrained at this location. The methods described herein can therefore potentially improve patient treatment by eliminating the need to induce VT, and by shortening procedure time.

## Conflicts of interest

The authors have no conflicts of interest.

## Figures and Tables

**Table 1:** Unidirectional Block and Isthmus Measurements.

Meas *a* (mm)	Meas *b* (mm)	Meas *c* (mm)	Meas *d* (mm)	Meas *d*/*c* (%)	Expt.
43.63	9.44	20.57	5.54	26.93	1
11.90	5.36	10.94	6.43	58.74	2
56.42	24.57	42.58	18.92	44.44	3
38.97	6.41	12.44	3.94	31.64	4
38.61	18.44	18.48	6.49	35.14	5
26.39	8.82	16.72	6.91	41.31	6
23.91	11.79	30.38	13.85	45.58	7
40.27	4.48	18.65	4.81	25.78	8
25.62	8.44	25.44	11.38	44.71	9
19.81	12.41	35.52	13.23	37.24	10
8.87	7.72	25.79	7.10	27.54	11
41.94	10.51	19.88	8.42	42.34	12
31.36	10.70	23.12	8.92	38.45	MN
14.18	5.73	9.34	4.49	9.69	SD

In this table, measurements described in the schematic of Fig. 3 are shown for the 12 canine postinfarction experiments. The first four measurements *a*, *b*, *c*, and *d* are distances between features in the infarct border zone while the last measurement is the ratio of *d*/*c* (percent). Meas: measurement. Expt.: experiment number. MN: mean. SD: standard deviation.

**Table 2:** Cycle Length.

PS (ms)	VT (ms)	Reexcitation (ms)	Expt.

160	183	171	1
160	197	159	2
170	217	113	3
160	182	140	4
140	167	133	5
160	174	167	6
170	177	103	7
180	185	145	8
175	221	156	9
175	220	184	10
170	202	147	11
155	163	193	12
164.6	190.7	150.9	MN
11.0	20.4	26.7	SD

In this table the coupling interval during premature stimulation (PS) leading to reentrant ventricular tachycardia (VT), and the mean cycle length of tachycardia over the first 10 cycles, are shown in the first two columns for all 12 canine postinfarction experiments. In the third column the reexcitation interval, described in detail in the text, is noted. PS: the premature stimulus coupling interval in milliseconds. VT: The mean cycle length during the first 10 beats of VT in milliseconds. Reexcitation: the reexcitation interval in milliseconds. Expt.: the experiment number. MN: mean. SD: standard deviation.
